# Disease, the Voluntary Hospitals, and Mr. Lloyd George

**Published:** 1911-10-28

**Authors:** James Crichton-Browne


### DISEASE, THE VOLUNTARY HOSPITALS, AND MR. LLOYD GEORGE.

By SIR JAMES CRICHTON-BROWNE, M.D., LL.D., F.R.S.

In that great play which Sir Herbert Beerbohm Tree has presented to us in so great, so weirdly poetical and mystically spectacular a manner the doctor is asked by Macbeth:

"Canst thou not minister to a mind diseased;
Pluck from the memory a rooted sorrow;
Raze out the written troubles of the brain;
And, with some sweet oblivious antidote,
Cleanse the stuff'd bosom of that perilous stuff
Which weighs upon the heart?"

the doctor's reply was:—

"Therein the patient
Must minister to himself."

And I venture to think that the doctor was quite wrong.

I am not going to contend that Lady Macbeth's state of melancholia and somnambulism was curable. It was perhaps too deep-rooted for poppy or mandragora or bromide of potassium, or any other sweet oblivious antidote; but of this I feel sure, that all those who are not doctors, but support the League of Mercy, are doing something to

"Raze out the written troubles of the brain,
. . . . . . .
Cleanse the stuff'd bosom of that perilous stuff
Which weighs upon the heart"; for they are contributing to soothe and alleviate the sufferings, anxieties, and sorrows of those who are smitten by maladies of many kinds, to banish dark shadows and vague apprehensions, and to plant hope—and there is no better restorative than hope—in hearts that are sunk in despair. I want you to remember that all bodily diseases have their mental and emotional accompaniments. We have the inextinguishable hopefulness and sanguine anticipations of consumption up to the very last, and we have the black despondency and anguish of cancer, aggravating its malignant career and perhaps hastening its tragic issue.

And not only has every disease its own mental and emotional accompaniments, but every disease is influenced by what I may call the moral atmosphere around it. That may be favourable or unfavourable to recovery, and it should be our aim to create a moral atmosphere that is cheering and beneficent; and it is no small part of the advantages conferred by our voluntary hospitals that they provide that atmosphere. Those brought to them from the squalid and noisome hovels of the poor, even from the necessarily narrow and dingy dwellings of the better class, find in their spacious and airy wards, in their spotless brightness, in their well-ordered tranquillity, in their nursing and scientific equipment, a sense of confidence in which they may possess and sustain their souls, an environment conducive to recovery. Their stuffed bosoms are to some extent cleansed of that perilous stuff—fears and forebodings that almost always weigh upon the hearts of those who are seriously ill, and especially of those who have operations to encounter.

No private charitable ministrations, however well devised and kindly they may be, can equal the salutary influence exerted by a first-class hospital on those admitted to its benefits—apart altogether from the medical and surgical skill that it makes available. No doubt the removal from the home, humble though it may be, is distressing and depressing enough, but the great and urgent longing of the sick is to get well as soon as possible, and the realisation of that longing seems to be brought immeasurably nearer by the hospital which induces the mental and emotional attitude calculated to give treatment the best chance. So decisive are the advantages offered by hospital organisation that some distinguished surgeons in these days decline to perform serious operations in the homes of even the affluent classes, and insist on the removal of the patient to a nursing home.

I have spoken of the general effect of the hospital atmosphere, but that is, of course, subsidiary—wholly subsidiary—to the medical and surgical skill placed at the service of their patients by our great public hospitals. That is unquestionably their transcendent merit, and constitutes them one of the glories of our country. Pray bear in mind that those hospitals to which you are asked to lend a helping hand do offer to the poorest medical and surgical treatment as good, as skilful, as can be commanded by the richest in the land.

It has been my lot to witness lately the same surgical operation performed on two girls, one the child of affluent parents, in a nursing home, the other, the child of working people, in one of our hospitals. The one operation cost the parents two or three hundred guineas, and the other operation cost the parents not a farthing, but the two operations were indistinguishable in any particular. The poor girl had every advantage enjoyed by the rich one in exquisite surgical dexterity, in aseptic precautions, in appliances, in nursing, and in after care.

And that is as it should be, for disease and danger level all, and the best treatment should be accessible to all; but that is only possible by the exertion of agencies like your League of Mercy. Our hospitals are, as I have said, one of the glories of our country, and are unsurpassed in the civilised world. But how have they attained that proud position? Why, by voluntary effort. Would it be prudent, then, to interfere in any way with the machinery that has secured such excellent results? Is it not clearly our policy to perfect that machinery as far as possible, and to continue to trust to it?

I do not hesitate to affirm that if the Government were to take over all our voluntary hospitals and provide fully the funds necessary for their maintenance, that would be a great national calamity. It would dry up broad streams of human sympathy that flow through the community at present, softening and fertilising as they flow; it would withdraw from our hospitals that intimate and all-pervading personal interest which at present vitalises them; it would make their organisation rigid and wooden instead of plastic and mobile as it is at present; it would ultimately tend to impair their usefulness as centres of medical science and teaching. Widows' mites are in this connection worth far more than Parliamentary grants, and if our charities in this country are, in the progress of a mechanical socialism, to be superseded by State aids, then before long we shall be making our orisons through a prayer-wheel like the Lamas of Thibet.

We should, I think, jealously guard and maintain our voluntary hospitals whole and unimpaired. They are good for the bodies and souls of our people. And those who appreciate their merits cannot, I think, regard without apprehension some dangers by which they are threatened. The National Insurance Bill, unless its provisions are considerably modified, will, it is to be feared, cripple our hospitals. That was no doubt far from the intention of the author of the Bill. Mr. Lloyd George has said that the hospitals of this country—great institutions built up through years of toil, sacrifice and anxiety—are essentially a part of the machinery of civilisation, and that he could not imagine the country allowing a single hospital to be closed. He did not expect that as a result of the Act voluntary subscriptions to hospitals would be in any degree diminished. But those who have had practical experience in such matters take a contrary view and believe that while the Act will largely increase the demands made on hospitals, by the wives and children of wage-earners and by persons insured under it requiring special or operative treatment, and while it will impose a new and heavy burden upon them by the insurance of nurses, clerks, porters and servants, it will to a considerable amount cut off those annual subscriptions on which our hospitals largely subsist.

You of the League of Mercy who are engaged in collecting subscriptions must, I think, realise that employers of labour who are called upon to pay *3d.* a week for each employee in their service, that professional and trading classes who have to pay thirteen shillings a year for the insurance of each domestic servant, and that working men who have a penny a week deducted from their wages, will not be disposed to contribute as liberally as heretofore to the support of institutions providing merely a part of that medical service for which they are taxed.

Mr. Lloyd George has supreme faith in the goodness of human nature, and believes that as the Workman's Compensation Act, which it was predicted would injure hospitals and charities, has not done so, so the National Insurance Bill if it becomes an Act will prove inocuous. Employers and members of Friendly Societies will, he believes, go on subscribing as before. But I think you must have felt that there has been increased stringency and difficulty in keeping up subscriptions even in the metropolis in recent years. There must be a point where increased taxation by encroaching on the available margin of income necessitates a reduction in eleemosynary expenditure. Honesty comes before charity, and a man must pay his way.

Mr. Lloyd George admits that his Bill will cause a temporary irritation. The risk is that it may induce a permanent hardness of heart.

It is to be hoped, therefore, that means will be found to protect our voluntary hospitals from the losses with which they are menaced. The question is a very complex one, and I cannot discuss it here, but it seems to me that our hospitals should be placed on the same footing as the sanatoria for consumptives which it is proposed to provide—apparently on much too lavish a scale—and that Local Health Committees should be empowered to make arrangements with hospitals for the treatment of insured persons entitled to institutional benefit under the Act. Hospitals may properly be paid for work done, and this may be accomplished without any interference with their voluntary character, and without introducing into their management any Governmental or Friendly Society control. A scheme of this kind on a *pro rata* basis would relieve our voluntary hospitals from the embarrassments that threaten them and maintain intact their independent character.

But whatever may be the outcome of the National Insurance Bill, our voluntary hospitals will stand in as great need as ever of legacies, donations and subscriptions, and the assistance of the League of Mercy will be as necessary as ever. You have not to relax but to redouble your efforts. The calls on our hospitals are increasing, the cost of nursing and of treatment is rising, and funds are urgently required.

